# Control of Love waves by resonant metasurfaces

**DOI:** 10.1038/s41598-018-25503-8

**Published:** 2018-05-08

**Authors:** Antonio Palermo, Alessandro Marzani

**Affiliations:** Department of Civil, Chemical, Environmental and Materials Engineering - DICAM, University of Bologna, Bologna, 40136 Italy

## Abstract

Metasurfaces of mechanical resonators have been successfully used to control in-plane polarized surface waves for filtering, waveguiding and lensing applications across different length scales. In this work, we extend the concept of metasurfaces to anti-plane surface waves existing in semi-infinite layered media, generally known as Love waves. By means of an effective medium approach, we derive an original closed-form dispersion relation for the metasurface. This relation reveals the possibility to control the Love waves dispersive properties by varying the resonators mechanical parameters. We exploit this capability to manipulate the metasurface refractive index and design two gradient index (GRIN) metalenses, i.e. a Luneburg lens and a Maxwell lens. We confirm the performance of the designed lenses using full 3D finite element simulations. Our work demonstrates the possibility of realizing wave control devices for anti-plane waves.

## Introduction

The study of surface waves in solid materials is a classical topic of elastodynamics which still attracts the interest of numerous researchers from different disciplines such as geophysics, geotechnical engineering, solid-state physics, material science, electronic engineering, among the others. The interest arises from the multiple methodologies/technologies which utilize surface waves, as non-invasive testing for material characterization^1,2^, or filters, delay lines and transformers for electronic devices^3^. In addition, surface waves generated during an earthquake and guided by the topography and/or stratigraphy of the earth’s crust are often the cause of significant damages in buildings and infrastructures and hence are of paramount importance in seismology and earthquake engineering.

Surface waves in semi-infinite elastic media can be grouped in two distinct classes: (i) in-plane waves, which exist regardless of the medium stratigraphy (e.g. the Rayleigh waves in homogeneous half-spaces) and (ii) anti-plane waves, which can propagate only through inhomogeneous or layered materials. Love waves, which are the most known type of anti-plane waves, are guided modes traveling at the free surface of a soft elastic layer “welded” to a more rigid substrate. Their dispersive properties have been originally formalized by A. E. H. Love^4^ and later discussed in geophysical studies^5,6^, researches on piezoelectric layered media^7,8^ and applications in microsensing devices^9^.

Recently, the development of a new class of artificial composites, named elastic metamaterials, has opened unprecedented possibilities in the control of elastic waves. Elastic metamaterials utilize resonant particles or structures of sub-wavelength dimensions to provide unique dynamic properties to the medium. Frequency band-gaps, dynamic anisotropicity, negative effective mass and/or elastic moduli, negative refractive index, are just a few examples of these peculiar dynamic properties which are exploited to design and realize wave filters, waveguides, lenses and cloaking devices^10–12^.

Within the broad class of metamaterials, a “metasurface” consists in a periodic or random arrangement of resonant pillars/oscillators or resonant inclusions at the free surface of an elastic media, specifically designed to control surface waves^13^. Recent studies have shown how metasurfaces can control in-plane waves in half-spaces and plates^14–20^. For instance, vertical resonators have been used to inhibit the propagation of Rayleigh waves by converting them into shear bulk waves, paving the way for a novel class of seismic isolation devices^14,15^. Resonant micro-pillars have been proved to guide surface waves along specific paths in the form of hybridized modes with potential applications for surface acoustic wave devices^16,17^. Similarly, metasurfaces of resonant rods at the free surface of an elastic plate have been designed to control the dispersive properties of flexural waves for waveguiding, cloaking and lensing^18–20^. On the contrary, to the best of our knowledge, the interaction between anti-plane waves in layered media, e.g. Love waves, and metasurfaces of resonators has not been investigated yet.

For this reason, here we study the dynamics of a metasurface of horizontal mechanical oscillators coupled to anti-plane Love waves. We derive an original analytical dispersion relation for the metasurface which unveils the possibility to manipulate the Love waves dispersive properties. We support the analytical study with finite element (FE) simulations and Bloch wave theory to confirm the predicted dispersive properties. Our study is complementary to the theoretical background provided in recent works^13,21^ for the design of metasurfaces interacting with Rayleigh and flexural waves, and paves the way to the variety of applications already discussed for in-plane waves. Here, as an example, we show how the derived dispersion law can be used to design two gradient-index (GRIN) metalenses (a Luneburg lens and a Maxwell lens) for Love waves control. The work is organized as follows. First, we derive analytically and numerically the dispersive properties of the metasurface. The analytical model uses an effective medium approach assuming dimensions and distances between the resonators much smaller than the Love wavelength. This approach allows us identifying a single dimensionless parameter, here labeled as “*F*”, which controls the strength of interaction between Love waves and resonators. Additionally, we employ a FE numerical model to extract the metasurface dispersion curve and confirm the analytical findings using numerical simulations. Then, we show how to tune the dimensionless parameter “*F*” to manipulate the metasurface refractive index and design metalenses for Love wave control. We utilize the analytical dispersion relation to design metasurfaces which match the refractive index profile of a Luneburg lens and a Maxwell lens. Finally, we employ full 3D FE harmonic analyses to simulate the wavefield of Love waves traveling thorough the designed metalenses and discuss possible fields of application of Love metasurfaces.

## Results

### Love waves interacting with a metasurface

#### Analytical dispersion relation using an effective medium approach

**Figure 1:**
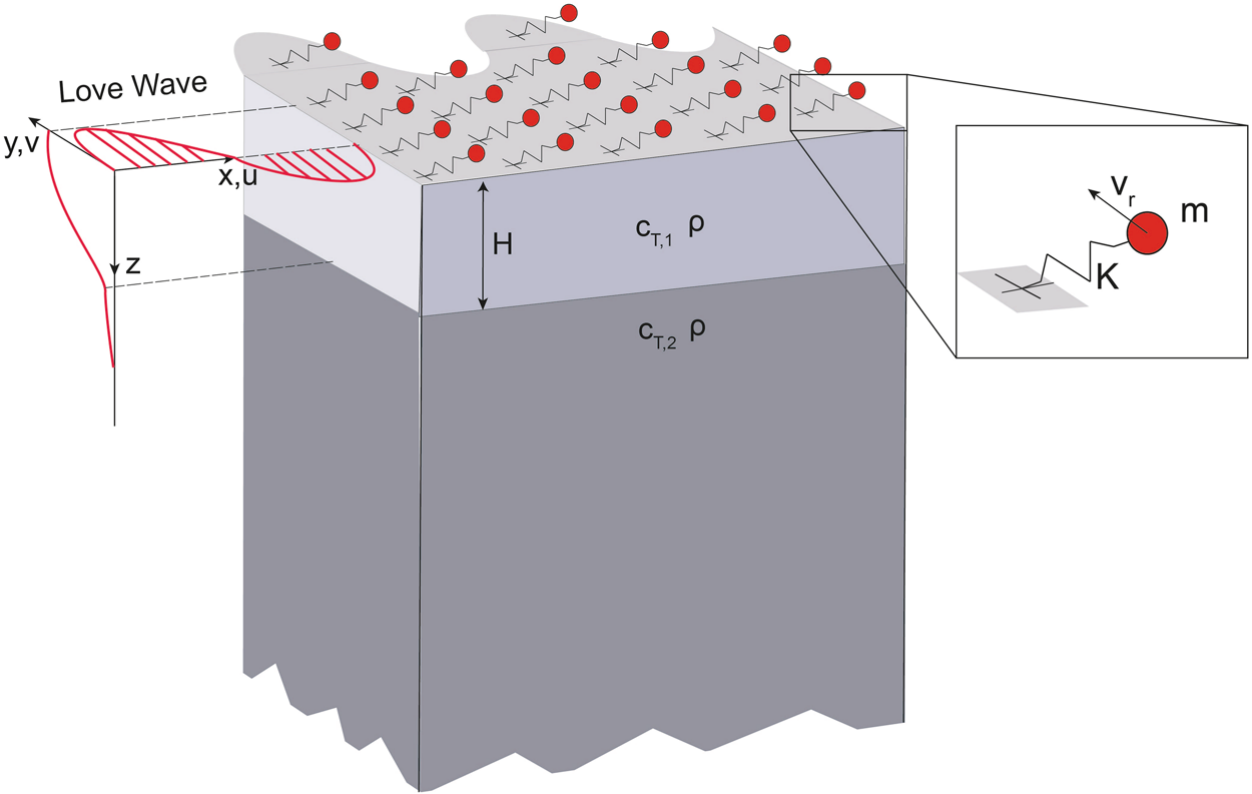
Schematic of Love waves interacting with an array of horizontal resonators.

We consider an elastic medium composed of a soft isotropic elastic layer of depth *H*, shear velocity *c*_*T*,1_ and density *ρ*, welded to a stiffer elastic isotropic half-space, of shear velocity *c*_*T*,2_ > *c*_*T*,1_ and density *ρ* (see Fig. 1). We analyze the interaction of plane harmonic Love waves of angular frequency *ω* and wavenumber *k*, polarized along the *y*-axis, with an array of horizontal mechanical resonators of resonance frequency ${\omega }_{r}=\sqrt{K/m}$, mass *m* and spring constant *K*, placed at the free-surface (*z* = 0) of the soft elastic layer. The spacing of the resonators is significantly shorter than the Love wavelength $\lambda =\frac{2\pi }{k}$ in the frequency range of interest allowing us to model the metasurface with an effective medium description^22–24^. Following this approach, the interaction between the layered medium and the metasurface is described by means of a uniform shear stress exerted by the metasurface over the elastic soft-layer:

1 $${\tau }_{yz,r}=\frac{P}{{A}_{r}}$$

In Eq. (1), *P* = *K*(*v*_1,0_ − *v*_*r*_) is the force exerted by each resonator, *v*_*r*_ is the horizontal displacement of the resonator mass along the *y*-axis, *v*_1,0_ is the horizontal displacement along the same direction at the resonator base and *A*_*r*_ = *A*_*t*_/*n* is the mean resonator area for a generic ensemble of *n* resonators distributed on a total area *A*_*t*_.

We obtain an analytical expression for the metasurface dispersion relation following a classical approach for Love waves^25^, and replacing the standard stress-free boundary condition at the medium surface with the metasurface tangential stress in Eq. (1) (see Supplementary Information for the detailed derivation):

2 $$\tan \,(k^{\prime} {s}_{1}H^{\prime} )=\frac{{s}_{2}^{\ast }}{{\alpha }^{2}{s}_{1}}\frac{(1-\frac{1}{k^{\prime} }\frac{F}{{s}_{2}^{\ast }}(\frac{{\omega }^{^{\prime} 2}}{1-{\omega }^{^{\prime} 2}}))}{(1+\frac{1}{k^{\prime} }\frac{{s}_{2}^{\ast }}{{s}_{1}^{2}}\frac{F}{{\alpha }^{4}}(\frac{{\omega }^{^{\prime} 2}}{1-{\omega }^{^{\prime} 2}}))}$$

where *ω*′ = *ω*/*ω*_*r*_ and *k*′ = *kc*_*T*,2_/*ω*_*r*_ are the dimensionless frequency and wavenumber. In Eq. (2), *α* = *c*_*T*,1_/*c*_*T*,2_, ${s}_{1}=\sqrt{\frac{{c}^{2}}{{c}_{T,1}^{2}}-1}$, ${s}_{2}^{\ast }=\sqrt{1-\frac{{c}^{2}}{{c}_{T,2}^{2}}}$, $H^{\prime} =\frac{H{\omega }_{r}}{{c}_{T,2}}$ is the non-dimensional depth of the soft layer and $F=\frac{m{\omega }_{r}}{{A}_{r}\rho {c}_{T,2}}$ is a dimensionless parameter which controls the interaction between Love waves and the oscillators. The same interaction parameter can be found in Ref.^24^ where it is shown how surface resonators can support the existence of shear horizontal waves in a half-space. Indeed, the dispersion relation of Ref.^24^ can be obtained from Eq. (2) for *H*′ = 0 (or *α* = 1), thus resulting in a special case of the present study. Solutions of the nonlinear Eq. (2) can be found numerically (see Methods).

**Figure 2:**
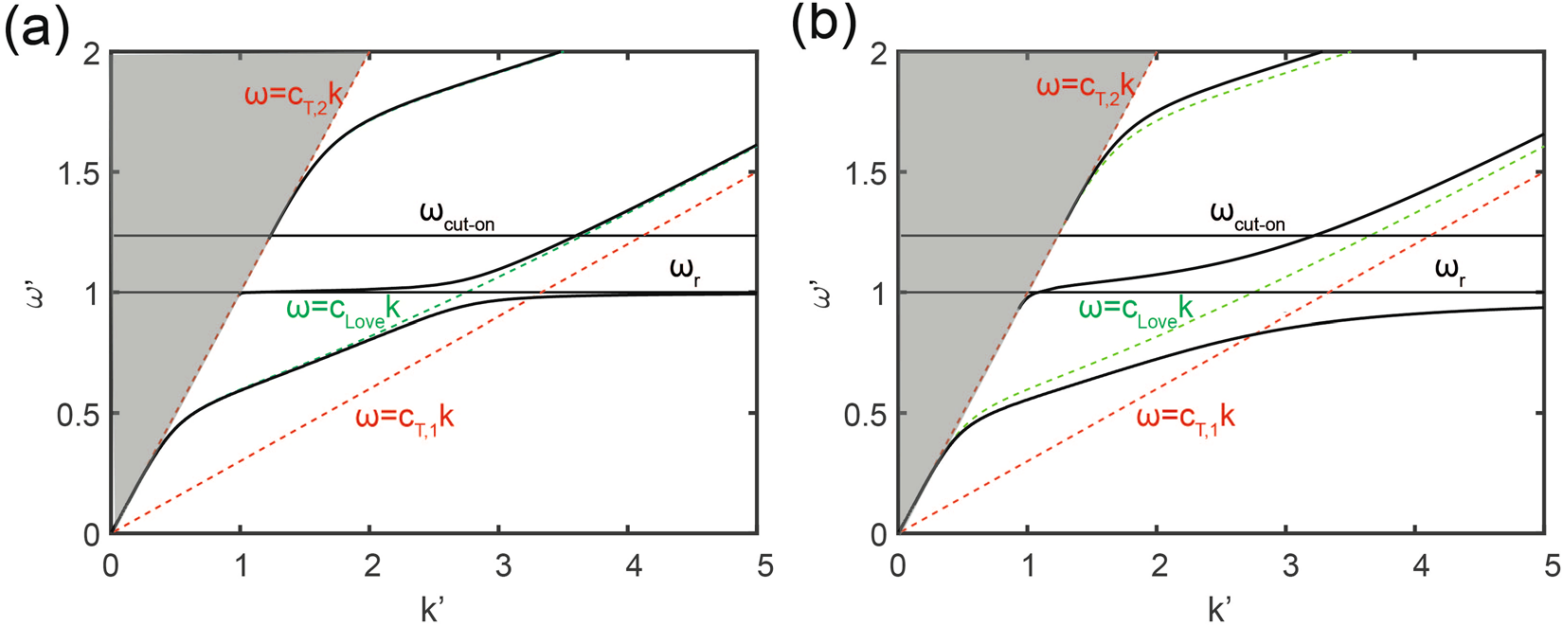
Dispersion relation of Love waves interacting with a metasurface of horizontal resonators (solid curves) as per Eq. (2) for (**a**) F = 0.005 and (**b**) F = 0.05. The dashed green curves correspond to the classical Love wave solutions (no resonators). The dashed red curves mark the bulk speeds in the upper layer (*c*_*T*,1_) and in the substrate (*c*_*T*,2_), respectively. The graded area identifies the “sound cone” where SH-bulk waves exist only in the half-space.

For any non-zero value of the dimensionless parameter *F*, the dispersion relation exhibits a classic “avoided crossing” behavior due to the hybridization of the fundamental Love mode (whose phase speed is denoted as *c*_*Love*_) with the metasurface resonance (see Fig. 2a, where *F* = 0.005, *α* = 0.3, *c*_*T*,2_ = 1, *ρ* = 1 and *H*′ = 0.848). In addition, we observe that the Love-metasurface coupling does not result in a significant frequency band-gap, which is instead characteristic of bulk^26^ and in-plane wave hybridizations^15,23,27^. Nonetheless, higher values of the coefficient *F*, achieved for example by means of higher resonant masses for a constant resonance frequency, lead to a stronger hybridization of the Love fundamental mode with flatter repelling branches observed around the resonance (see Fig. 2b, where *F* = 0.05). These dispersive features suggest the possibility to use the resonant metasurface to control the Love wave phase speed and eventually tune the metasurface refractive index.

#### Numerical dispersion verification using Bloch wave theory and FE modeling

**Figure 3:**
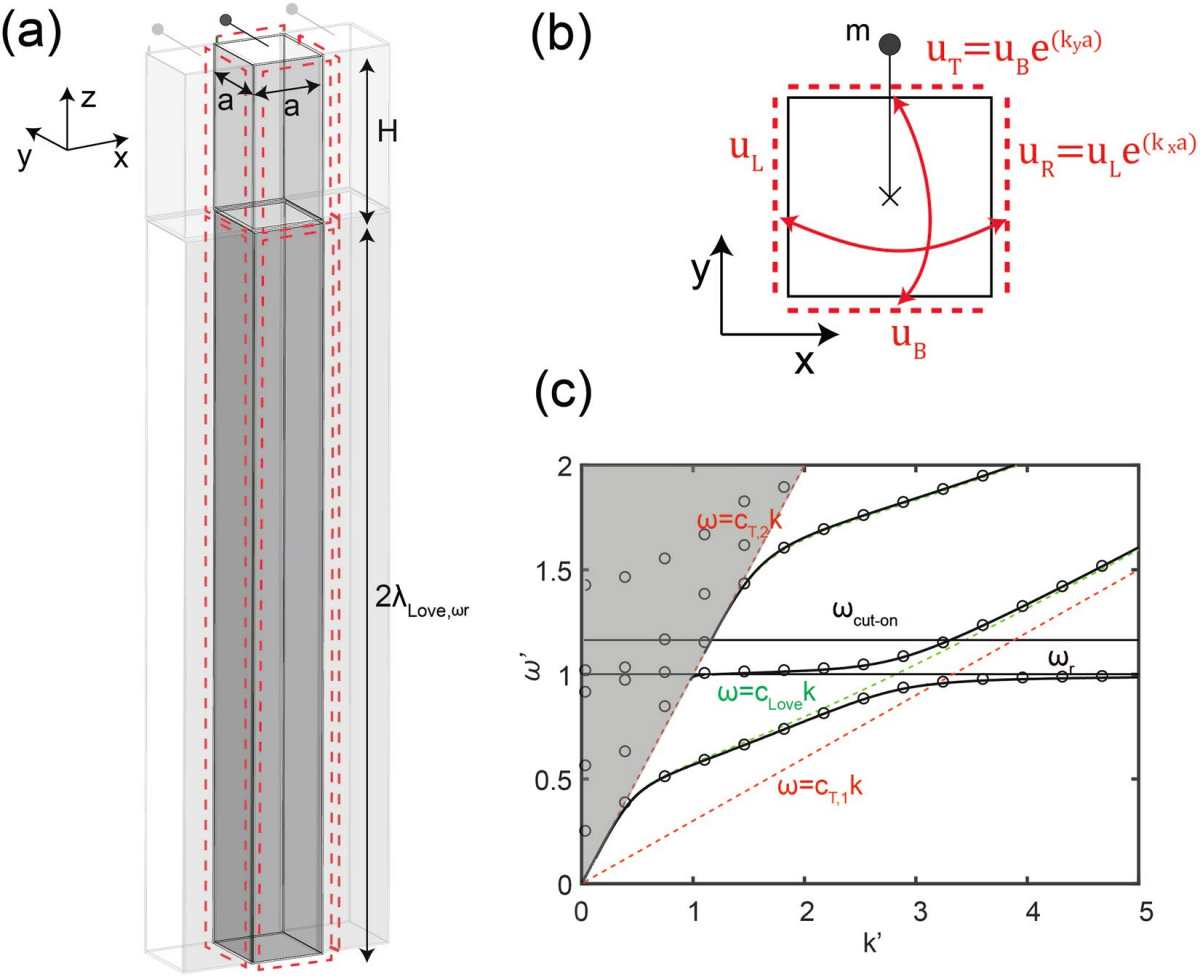
Numerical model for the metasurface dispersion relation. Schematic of the unit cell model: (**a**) 3D model (**b**) Plane view (x − y). Dashed red lines mark boundaries with corresponding Bloch-Floquet conditions. (**c**) Dispersion relation for Love waves propagating along the *x*-direction (*k*_*y*_ = 0) and interacting with a metasurface of horizontal resonators (along *y*-direction). The solution is calculated for *c*_*T*,2_ = 1, *α* = 0.3, *H*′ = 0.848, *F* = 0.01. Black dots are the solutions calculated with the FE model while continuous black lines are the analytical ones as per Eq. (2).

We employ full 3D finite element (FE) simulations to compute numerically the metasurface dispersion curves, assuming a periodic arrangement of the mechanical resonators and exploiting the Bloch-wave theory. To this aim, we imagine a square regular grid of surface horizontal resonators and identify the unit cell of such array (Fig. 3a). The in-plane area of the unit cell is *A*_*r*_ = *a* × *a* where $a=\frac{1}{30{\lambda }_{Love,{\omega }_{r}}}$ and ${\lambda }_{Love,{\omega }_{r}}=2\pi \frac{{c}_{Love}}{{\omega }_{r}}$ is the Love wavelength at the metasurface resonance. Such dimensions ensure that the interaction between the metasurface and the layered medium occurs in the sub-wavelength regime, as in the effective medium approach. The unit cell depth is set as $H+2{\lambda }_{Love,{\omega }_{r}}$ which allows to adequately represent the half-space condition. Each layer of the medium is modeled as a linear elastic material and the mechanical resonators are implemented using truss-mass elements of mass *m* and stiffness *K* oriented along the Love wave polarization (*y*-axis).

Fixed boundary conditions are used at the unit cell bottom surface, while Bloch-Floquet periodic boundary conditions are enforced at its lateral surfaces (see Fig. 3a,b). The use of Bloch-Floquet boundary conditions allows us to simulate the dynamics of an infinite array of horizontal resonator laying over the layered medium, modeling the single unit cell. We setup and solve the unit cell elastodynamic eigenproblem for different values of the wavenumber *k* along the *x* direction, obtaining the metasurface dispersion curves (details on the use of Bloch-Floquet boundary conditions and FE approaches for periodic/resonant systems can be found for example in Refs.^12,28,29^). The dispersion curves calculated for a system with *F* = 0.01, *α* = 0.3, *c*_*T*,2_ = 1 and *H*′ = 0.848, are provided in Fig. 3c. The FE eigensolutions, marked by the black circles, match exactly the analytical solution obtained from Eq. (2) and represented with continuous black lines. In addition, the FE model identifies plate-like modes in the sound cone, related to the finite dimensions of the model, which are not of interest for this study.

The implemented unit cell does verify the derived analytical dispersion law for the Love metasurface and can be used as the building block of 3D FE models to simulate the propagation of Love waves through metasurfaces.

### GRIN metalenses for Love waves control

#### Metalenses design procedure

We use a metasurface to manipulate the dispersive properties of Love waves and design gradient-index (GRIN) lenses for their control. A GRIN lens consists of a space region where specific material parameters (e.g. density, elastic moduli or waveguide dimensions) are finely tuned to obtain a position-dependent refractive index which guides the wavefront along desired trajectories^30,31^. Here, our strategy to design GRIN lenses with resonant metasurfaces, shortly referred to as “metalenses”, basically consists in varying the refractive index of the layered medium in a defined circular region, by varying the dimensionless parameters *F*, according to the refractive index law of the selected lens.

**Figure 4:**
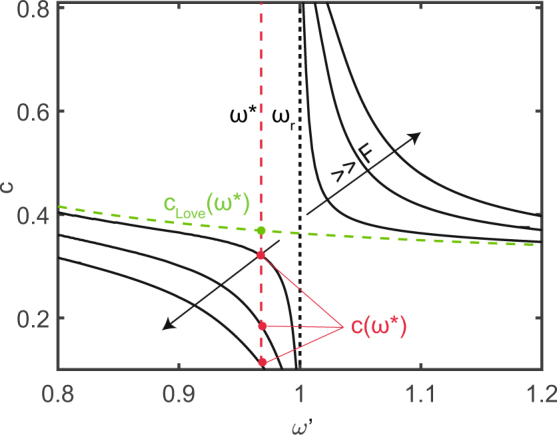
Phase velocities of the zero order Love waves hybridized by a metasurface of horizontal resonators (solid curves) as per Eq. (2) for *c*_*T*,2_ = 1, *α* = 0.3, *H*′ = 0.848, *F* = [0.005, 0.025, 0.05]. The dashed green curve corresponds to the zero order Love wave solution.

Indeed, around the metasurface resonance *ω*_*r*_, the hybridized Love waves phase speed *c* can be largely modified by varying the resonator interaction coefficient *F* as can be seen in Fig. 4. Assuming the fundamental Love wave speed *c*_*Love*_(ω^*^) at a given frequency ω^*^ as the reference velocity for the layered medium, the refractive index between the layered medium and the metasurface is calculated as ${n}_{index}({\omega }^{\ast })=\frac{{c}_{Love}({\omega }^{\ast })}{c({\omega }^{\ast })}$. Since the lower branch of the hybridized mode presents phase velocities significantly smaller than the fundamental Love wave supported by the medium *c*(ω^*^) < *c*_*Love*_(ω^*^), we can easily obtain refractive indexes *n*_*index*_ > 1 and finely tune them over large ranges as needed for the design of GRIN lenses. Indeed, a similar procedure has been already used to design GRIN lenses for vertically polarized waves in plates^32^.

As a result, the design of a metalens requires only the definition of the interaction coefficient profile *F*(*r*/*R*) to obtain a desired refractive index profile *n*_*index*_(*r*/*R*). Here, *R* is the radius of the lens while *r* is the radial position within the lens (0 ≤ *r* ≤ *R*).

**Figure 5:**
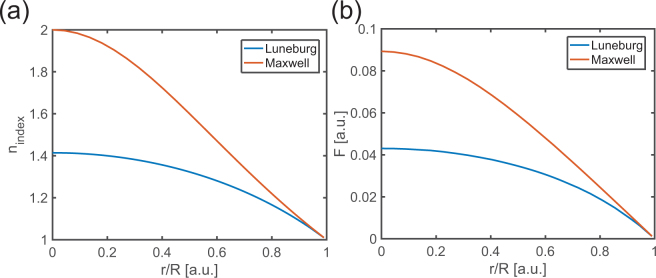
(**a**) Refractive index profile of the Luneburg lens and the Maxwell lens. (**b**) Calculated interaction coefficient profile for a Luneburg lens and a Maxwell lens.

We consider two specific GRIN lenses, namely (i) the Luneburg lens and (ii) the Maxwell lens whose refractive index profiles are given in Table 1 and shown in Fig. 5a.

**Table 1:** Refractive index profile of the Luneburg lens and Maxwell lens.

Lens	Refractive index *n*_*index*_
Luneburg	${n}_{index}=\sqrt{2-\frac{{r}^{2}}{{R}^{2}}}$
Maxwell	${n}_{index}=\frac{2}{1+{r}^{2}/{R}^{2}}$

We discretize the radial coordinate *r* at *n* equally spaced positions *r* = [*r*_1_, *r*_2_, …, *r*_*n*_], with *r*_1_ = 0 and *r*_*n*_ = *R*, and calculate the corresponding refractive index *n*_*index*_ (*r*_*i*_) according to the Eqs. in Table 1. We set the target phase speed at the given working frequency of the metalens ω^*^ and radial coordinate *r*_*i*_ as $\mathop{c}\limits^{ \sim }({\omega }^{\ast },{r}_{i})=\frac{1}{{n}_{index}({r}_{i})}{c}_{Love}({\omega }^{\ast })$ and look for the interaction coefficient *F*(*r*_*i*_) as the zero of the function:

3 $${\rm{\Phi }}({\omega }^{\ast },F({r}_{i}))=c({\omega }^{\ast },F({r}_{i}))-\tilde{c}({\omega }^{\ast },{r}_{i})$$

where *c*(ω^*^, *F*(*r*_*i*_)) is the phase velocity calculated as per Eq. (2). Standard root-finding algorithm (e.g. the Bisection Method) can be used to solve the nonlinear Eq. (3).

We remark that a variation of the interaction coefficient *F* can be obtained either by changing proportionally the mass *m* and stiffness *K* of the resonator (i.e. keeping the metasurface resonance *ω*_*r*_ constant) or by changing the resonator resonance *ω*_*r*_. Here we adopt the first strategy, to preserve the same non-dimensional *ω*/*ω*_*r*_ description of the metasurface dispersive properties over the lens surface.

Finally, the values of the interaction coefficient at the discrete locations *F*(*r*_*i*_/*R*) are used to fit a polynomial function in order to obtain a continuous description of the interaction coefficient over the lens domain *F*(*r*/*R*). The interaction coefficient profiles *F*(*r*/*R*) calculated according the above described procedure for a layered medium with velocities *c*_*T*,2_ = 1, *α* = 0.3, *H*′ = 0.848, ω^*^ = 0.89*ω*_*r*_ for both a Luneburg and a Maxwell lens are shown in Fig. 5b.

#### Metalenses FE simulations

**Figure 6:**
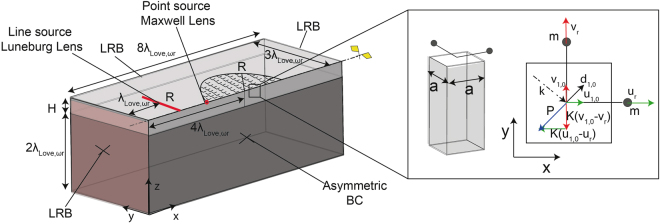
Schematic of the 3D FE model. In the inset, the details of the double-resonator arrangement used for the metalens model.

We use full 3D FE simulations to test the designed Luneburg and Maxwell lenses. The FE model, shown in Fig. 6, has a length of 8 ${\lambda }_{Love,{\omega }_{r}}$ and a width of 3 ${\lambda }_{Love,{\omega }_{r}}$. As for the unit cell simulations, we set the 3D model depth as *H* + $2{\lambda }_{Love,{\omega }_{r}}$, where *H* is the depth of the soft layer and $2{\lambda }_{Love,{\omega }_{r}}$ the depth of the stiffer medium, sufficient to ensure the correct half-space representation in the frequency range of interest. Asymmetric boundary conditions allows modeling a double-width domain with a reduced computational effort. In addition, Low-Reflective Boundary (LRB) conditions are used to minimize boundary reflections in this finite-dimension model. The metalens is centered at coordinates $x=4{\lambda }_{Love,{\omega }_{r}}$, *y* = 0 and has a radius *R* = $1.5{\lambda }_{Love,{\omega }_{r}}$. The resonators, modeled using truss-mass elements as in the unit cell simulations, are arranged within the metalens circular domain on a square grid with unit cell dimensions *A*_*r*_ = *a* × *a*, where $a=\frac{{\lambda }_{Love,{\omega }_{r}}}{30}$.

The mass and stiffness of the resonators along the radial coordinate are defined according to the relationships:

4 $$m(r)=\frac{F(r/R){A}_{r}\rho {c}_{T,2}}{{\omega }_{r}}\,K(r)=m(r){\omega }_{r}^{2}$$

were *F*(*r*/*R*) is given in Fig. 5b. Since each truss-mass element has a mono-axial behavior, for each unit cell we utilize a couples of identical resonators in an orthogonal configuration to reconstruct the force P orthogonal to the wavefront direction, which varies along the metalens domain (see also inset Fig. 6). As such, the surface load P reads:

5 $$P=\sqrt{{P}_{x}^{2}+{P}_{y}^{2}}=-\,K\sqrt{{({u}_{1,0}-{u}_{r})}^{2}+{({v}_{1,0}-{v}_{r})}^{2}}=K\frac{{\omega }^{2}}{{\omega }_{r}^{2}-{\omega }^{2}}{d}_{1,0}$$

where ${d}_{1,0}=\sqrt{{V}_{1,0}^{2}+{U}_{1,0}^{2}}$ is the Love wave amplitude at the resonator base (see the Supplementary Information for details on *V*_1,0_).

Here, by assuming a substrate shear velocity *c*_*T*,2_ = 1000 m/s and a resonator frequency *f*_*r*_ = *ω*_*r*_/(2*π*) = 4.5 Hz, the non-dimensional set of parameters is easily adapted to a realistic geophysical scenario (*c*_*T*,1_ = *αc*_*T*,2_ = 300 m/s, *f* ^*^ = ω^*^/(2*π*) = 0.89*ω*_*r*_/(2*π*) = 4.0 Hz, *H* = *H*′*ω*_*r*_/*c*_*T*,2_ = 30 m) for the design of meter-scale (${\lambda }_{Love,{\omega }_{r}}=93\,{\rm{m}}$) metasurfaces and metalenses.

**Figure 7:**
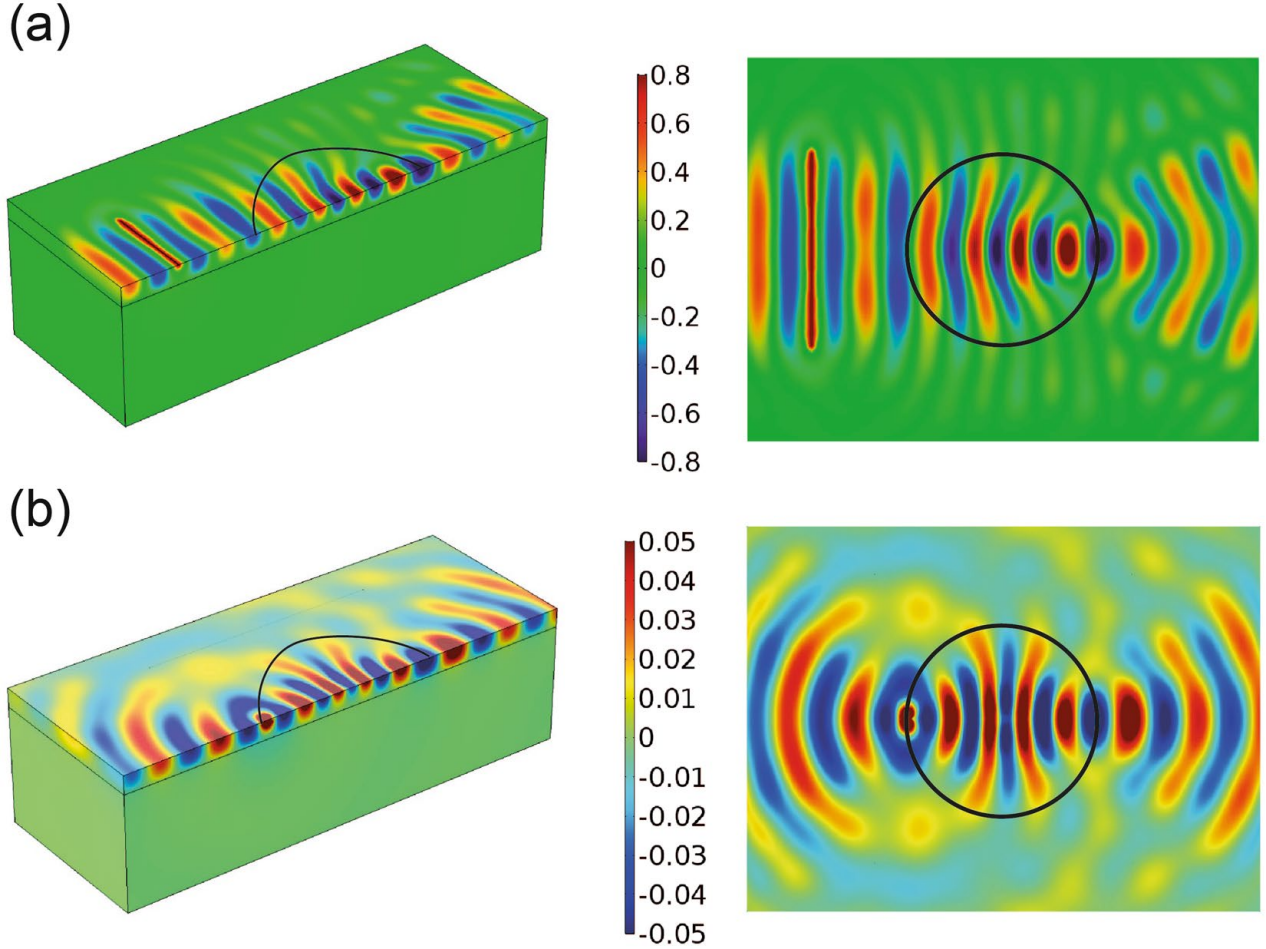
FE simulation of the design GRIN lenses. (**a**) Displacement field (*v* component) of harmonic Love waves (*f* ^*^ = 4.0 Hz) traveling through the Luneburg lens (**b**) Displacement field (*v* component) of a harmonic point source (*f* ^*^ = 4.0 Hz) focused by a Maxwell fish-eye lens.

The metalenses performances are evaluated using harmonic simulations at the lens working frequency *f* ^*^ = 4.0 Hz. For the Luneburg lens, we use a harmonic imposed displacement (*u* = 0, *v* = *V*_*imp*_*sin*(ω^*^t), *V*_*imp*_ = 1) over a line-source, placed at a distance of $x={\lambda }_{Love,{\omega }_{r}}$ from the domain boundary. The displacement field along the *y*-direction as obtained from the harmonic simulation is shown in Fig. 7a. The plane wave generated by the line-source is focused by the designed Luneburg lens at its border. The planar view, obtained by mirroring the computed wavefield, allows fully appreciating the expected focusing behavior of the lens.

For the Maxwell lens, instead, we use a point source of prescribed hamonic displacements (*u* = *v* = *V*_*imp*_ sin (ω^*^t)) placed at the border of the lens ($x=3{\lambda }_{Love,{\omega }_{r}}$, $y=1/100{\lambda }_{Love,{\omega }_{r}}$). Figure 7b shows the simulated wavefield where the point source excited at border of the lens is refocused at the opposite point.

We remark that the metasurface refractive indexes have been theoretically predicted from the dispersion law assuming an infinite array of resonators with a constant interaction coefficient *F*. This assumption is clearly not fulfilled by the metalens resonator arrangement which has finite dimensions and a smoothly varied interaction coefficient over the radial coordinate. However, the results provided by the numerical simulations suggest that the considered variation of the interaction coefficient do not significantly compromise the local interaction of the single resonators. Indeed, similar conclusions were drawn for the design of metalenses for flexural waves in plates^32^.

## Discussion

In summary, we have studied the interaction between an array of surface mechanical resonators, here shortly referred as metasurface, and Love waves, horizontal shear waves which travel confined by a soft elastic layer over a stiffer substrate. By deriving an original dispersion relation for this system, we have unveiled the ability of a metasurface to manipulate the Love wave dispersive properties. In particular, we have identified a dimensionless parameter *F* which controls the strength of interaction between the mechanical resonators and the Love waves. By tuning this interaction coefficient we can finely control the Love wave phase speed and hence the metasurface refractive index. This ability has been used to design GRIN metalenses for Love wave redirection. The refractive index profiles of a Luneburg lens and a Maxwell lens have been obtained by adjusting locally the interaction coefficient of the metasurface over the lens domain. The computed numerical results provide the expected lensing behavior and confirm the dispersion model used to predict the metasurface refractive index.

We expect that the theoretical framework developed in this paper will serve as a guide for the design of metasurfaces to control Love waves at multiple length scales, from microsensing applications to large-scale seismic isolation devices.

## Methods

### Dispersion relation: roots extraction

The roots of the Love-metasurface dispersion relations displayed in Fig. 2(a,b) are calculated by searching for the real wavenumber *k*′ which minimizes the expression:

6 $$D(\omega ^{\prime} ,k^{\prime} )=\,\tan \,(k^{\prime} {s}_{1}H^{\prime} )-\frac{{s}_{2}^{\ast }}{{\alpha }^{2}{s}_{1}}\frac{(1-\frac{1}{k^{\prime} }\frac{F}{{s}_{2}^{\ast }}(\frac{{\omega }^{^{\prime} 2}}{1-{\omega }^{^{\prime} 2}}))}{(1+\frac{1}{k^{\prime} }\frac{{s}_{2}^{\ast }}{{s}_{1}^{2}}\frac{F}{{\alpha }^{4}}(\frac{{\omega }^{^{\prime} 2}}{1-{\omega }^{^{\prime} 2}}))}$$

for a given 0 < *ω*′ < 2. The real wavenumber *k*′ is sought within the domain *k*′ = [0, 20]. The minimization procedure is implemented in a MATLAB script.

### Numerical simulations

FE simulations of the unit cell and of the metalenses have been carried out using the commercial software COMSOL Multiphysics.

For the unit cell FE model discretization, we use a mesh of tetrahedral elements (quadratic lagrangian shape functions), with a minimum dimension ${d}_{{\min }}\le {\lambda }_{Love,{\omega }_{r}}/30$ which ensures an accurate calculation of the dispersion properties in the frequency range of interest. The FE eigensolutions are found using a direct solver (MUMPS).

Similarly, for the metalense FE model discretization, we use a mesh of tetrahedral elements (quadratic lagrangian shape functions), with a minimum dimension ${d}_{min}\le {\lambda }_{Love,{\omega }_{r}}\mathrm{/30}$ within the metalense region and a maximum dimension ${d}_{max}={\lambda }_{Love,{\omega }_{r}}\mathrm{/10}$ in the outer region which ensure an accurate description of the wave propagation phenomena in both the domains.

### Data availability

All data generated or analysed during this study are included in this published article (and its Supplementary Information file).

## Electronic supplementary material

Supplementary Information: Control of Love waves by resonant metasurfaces
