# Supplementary material for: Control of Love waves by resonant metasurfaces
Source: Sci Rep. 2018 May 8;8:7234. doi: 10.1038/s41598-018-25503-8 (PMC5940672; doi:10.1038/s41598-018-25503-8)
Supplement: Supplementary file 1 — Supplementary Information: Control of Love waves by resonant metasurfaces [file 41598_2018_25503_MOESM1_ESM.pdf]

Supplementary Information: Control of Love waves by resonant metasurfaces

Antonio Palermo[1] **and Alessandro Marzani**[1,*]

[1]University of Bologna,Department of Civil, Chemical, Environmental and Materials Engineering - DICAM, Bologna, 40136, Italy
[*]alessandro.marzani@unibo.it

DERIVATION OF THE DISPERSION RELATION FOR LOVE WAVES COUPLED WITH A METASURFACE OF HORIZONTAL RESONATORS

We consider the propagation of Love waves, i.e. shear horizontal (SH) waves guided by a soft isotropic elastic layer of depth H (i.e. layer 1) welded to a stiffer elastic isotropic half-space (i.e. layer 2) (see Fig. 1 in the main text). Without loss of generality, we assume the two layers to have an identical density $\rho_1 = \rho_2 = \rho$ and two different shear velocity $c_{T,1}$ and $c_{T,2}$, with $c_{T,1} = \alpha c_{T,2}$ and $\alpha < 1$. We analyze the interaction of Love waves with an array of horizontal mechanical resonators of mass m and spring constant K placed at the free-surface $(z = 0)$ of the soft elastic layer. Following a classical approach for Love waves dispersion analysis, we consider plane harmonic waves propagating along the x-axis, polarized along the y-axis, with their amplitude varying with the depth z. We denote by ω the wave angular frequency, and by k the wavenumber along the direction of propagation, with the same propagation velocity $c = \omega/k$ in the layer and in the half-space. We assume the displacement vectors in the layer, $\mathbf{u_1}$, and in the half-space, $\mathbf{u_2}$, of the form:

$$\mathbf{u_i} = [u_i, v_i, w_i] \quad i = 1,2 \tag{1}$$

with:

$$u_1 = u_2 = w_1 = w_2 = 0 \tag{2}$$

and:

$$\begin{aligned} v_1(x,z,t) &= V_1(z)e^{i(\omega t - kx)} \\ v_2(x,z,t) &= V_2(z)e^{i(\omega t - kx)} \end{aligned} \tag{3}$$

The displacements v_1, v_2 in Eq. (3) must satisfy the scalar equation of motion for transverse shear waves in both the isotropic layers:

$$\nabla^2 v_i = \frac{1}{c_{T,i}^2}\frac{\partial^2 v_i}{\partial t^2} \quad i = 1,2 \tag{4}$$

To this aim, the transverse displacements of Eq. (3) take the form:

$$\begin{aligned} v_1(x,z,t) &= [Ae^{-iks_1 z} + Be^{iks_1 z}]e^{i(\omega t - kx)} \\ v_2(x,z,t) &= [Ce^{-iks_2 z} + De^{iks_2 z}]e^{i(\omega t - kx)} \end{aligned} \tag{5}$$

with:

$$s_1 = \sqrt{\frac{c^2}{c_{T,1}^2} - 1} \quad \text{and} \quad s_2 = \sqrt{\frac{c^2}{c_{T,2}^2} - 1} \tag{6}$$

As regards to the metasurface, the equation of motion of each horizontal resonator reads:

$$m\ddot{v}_r = K(v_{1,0} - v_r) \tag{7}$$

where $v_r(x,t) = V_r e^{i(\omega t - kx)}$ is the horizontal displacement of the resonator mass along the y-axis, and $v_{1,0} = v_1(x,0,t) = V_{1,0} e^{i(\omega t - kx)}$ is the horizontal displacement along the same direction at the base of the resonator ($z = 0$).
Expliciting v_r, $v_{1,0}$ in Eq. (7), we evaluate the amplitude of the resonator motion V_r as:

$$V_r = \frac{\omega_r^2}{\omega_r^2 - \omega^2} V_{1,0} \tag{8}$$

where $\omega_r = \sqrt{K/m}$ is the resonator natural frequency.
We assume the spacing of the resonators to be significantly shorter than the Love's wavelength $\lambda = \frac{2\pi}{k}$ in the frequency range of interest. This allows us defining a uniform shear stress exerted by each resonator over the layer surface $\tau_{zy,r}$:

$$\tau_{yz,r} = \frac{P}{A_r} = -\frac{K}{A_r}\left(\frac{\omega^2}{\omega_r^2 - \omega^2}\right) V_{1,0} e^{i(\omega t - kx)} = -\frac{m\omega_r^2}{A_r}\left(\frac{\omega^2}{\omega_r^2 - \omega^2}\right) V_{1,0} e^{i(\omega t - kx)} \tag{9}$$

where $P = K(v_{1,0} - v_r)$ is the force exerted by the resonator and $A_r = A_t/n$ is the mean resonator area for a generic ensemble of n resonators distributed on a total area A_t. In doing so, we provide an effective medium description of the metasurface, which ignores the particular arrangement (periodic or random) of the resonators.
A this stage, we can derive the metasurface dispersion law starting from the following set of boundary conditions:

$$\tau_{zy,1} = \tau_{zy,r} \qquad \text{at} \quad z = 0, \tag{10}$$

$$v_1 = v_2 \qquad \text{at} \quad z = H, \tag{11}$$

$$\tau_{zy,1} = \tau_{zy,2} \qquad \text{at} \quad z = H, \tag{12}$$

$$v_2(x,z,t) \to 0 \qquad \text{at} \quad z \to \infty \tag{13}$$

where Eq. (10) enforces the resonator shear stress at the surface, Eqs. (11) and (12) impose the continuity of displacements and stresses at the layers interface, respectively, while Eq. (13) guarantees surface-bounded solutions of the form:

$$v_2(x,z,t) = C e^{-ks_2^* z} e^{i(\omega t - kx)}, \quad D = 0 \tag{14}$$

for any $c < c_{T,2}$, where:

$$s_2^* = \sqrt{1 - \frac{c^2}{c_{T,2}^2}} \tag{15}$$

By combining Eqs. (10)-(12), linear elastic constitutive relations $\tau_{zy,i} = \mu_i \frac{\partial v_i}{\partial z}$ $(i = 1,2)$, where $\mu_i = \rho c_{T,i}^2$ is the layer shear modulus, and the assumed displacement fields in Eq. (5) and Eq. (14), we obtain the following system of equations:

$$\begin{bmatrix} b_1 & b_2 \\ c_1 & c_2 \end{bmatrix} \cdot \begin{bmatrix} B \\ C \end{bmatrix} e^{i(\omega t - kx)} = \begin{bmatrix} 0 \\ 0 \end{bmatrix} \tag{16}$$

with:

$$b_1 = \left[\frac{i\mu_1 k s_1 - \frac{m\omega_r^2}{A_r}\left(\frac{\omega^2}{\omega_r^2 - \omega^2}\right)}{i\mu_1 k s_1 + \frac{m\omega_r^2}{A_r}\left(\frac{\omega^2}{\omega_r^2 - \omega^2}\right)} e^{iks_1 H} + e^{-iks_1 H}\right] \tag{17}$$

$$b_2 = -e^{-ks_2^* H} \tag{18}$$

$$c_1 = \left[\frac{i\mu_1 k s_1 - \frac{m\omega_r^2}{A_r}\left(\frac{\omega^2}{\omega_r^2 - \omega^2}\right)}{i\mu_1 k s_1 + \frac{m\omega_r^2}{A_r}\left(\frac{\omega^2}{\omega_r^2 - \omega^2}\right)} i\mu_1 k s_1 e^{iks_1 H} - i\mu_1 k s_1 e^{-iks_1 H}\right] \tag{19}$$

$$c_2 = \mu_2 k s_2^* e^{-ks_2^* H} \tag{20}$$

The system of Eq. (16) admits non-trivial solutions when its determinant is null. This condition leads to the metasurface dispersion relation:

$$\tan(ks_1H) = \frac{\mu_2 s_2^*}{\mu_1 s_1} \frac{\left(1 - \frac{1}{k}\frac{m\omega_r^2}{A_r\mu_2 s_2^*}\left(\frac{\omega^2}{\omega_r^2-\omega^2}\right)\right)}{\left(1 + \frac{1}{k}\frac{\mu_2}{\mu_1^2}\frac{s_2^*}{s_1^2}\frac{m\omega_r^2}{A_r}\left(\frac{\omega^2}{\omega_r^2-\omega^2}\right)\right)} \tag{21}$$

We remark that from Eq. (21), one can easily recover the classical Love dispersion relation for a null mass of the resonator:

$$\text{for} \quad m = 0 \quad \rightarrow \quad \tan(ks_1H) = \frac{\mu_2 s_2^*}{\mu_1 s_1} \tag{22}$$

In addition, we remind that Love waves are dispersive, i.e. their phase velocity c depends on the wave angular frequency ω, and that multiple Love modes are supported by a soft layer over an half space, namely a fundamental or zero order mode, which exists at $\omega = 0$, and higher order solutions whose cut-on frequencies are given by the equation[1]:

$$\omega_{cut-on,n} = \frac{n\pi}{H\sqrt{1/c_{T,1}^2 - 1/c_{T,2}^2}} \tag{23}$$

In this study, we restrict our interest to the interaction of mechanical resonators with the first fundamental Love mode, i.e. $\omega_r < \omega_{cut-on,1}$. Introducing the dimensionless parameters $\omega' = \omega/\omega_r$, $k' = kc_{T,2}/\omega_r$, $H' = \frac{H\omega_r}{c_{T,2}}$, $F = \frac{m\omega_r}{A_r\rho c_{T,2}}$ in Eq. (21), the dispersion relation in Eq. 2 of the main text is found.

References

1. Aki, K. & Richards, P. G. *Quantitative seismology* (2002).
